# Metagenomic Survey Reveals More Diverse and Abundant Antibiotic Resistance Genes in Municipal Wastewater Than Hospital Wastewater

**DOI:** 10.3389/fmicb.2021.712843

**Published:** 2021-08-30

**Authors:** Dengwei Zhang, Ye Peng, Chak-Lun Chan, Hilda On, Hogan Kok-Fung Wai, Sandeep Singh Shekhawat, Akhilendra Bhushan Gupta, Alok Kumar Varshney, Rungtip Chuanchuen, Xudong Zhou, Yankai Xia, Suisha Liang, Keiji Fukuda, Krishna Mohan Medicherla, Hein M. Tun

**Affiliations:** ^1^School of Public Health, Li Ka Shing Faculty of Medicine, University of Hong Kong, Hong Kong SAR, China; ^2^HKU-Pasteur Research Pole, University of Hong Kong, Hong Kong SAR, China; ^3^Department of Civil Engineering, Malaviya National Institute of Technology, Jaipur, India; ^4^Department of Bioengineering and Biotechnology, Birla Institute of Technology, Jaipur, India; ^5^Department of Biotechnology and Bioinformatics, Birla Institute of Scientific Research, Mesra, Ranchi, India; ^6^Department of Veterinary Public Health, Faculty of Veterinary Science, Chulalongkorn University, Bangkok, Thailand; ^7^Institute of Social and Family Medicine, School of Medicine, Zhejiang University, Hangzhou, China; ^8^School of Public Health, Nanjing Medical University, Nanjing, China

**Keywords:** antibiotic resistance, antibiotic resistance genes, biocide/metal resistance genes, hospital wastewater, municipal wastewater

## Abstract

Alongside antibiotic resistance, co-selection of antibiotics, biocides, and metal resistance is a growing concern. While hospital wastewater is considered a hotspot for antibiotic-resistant bacteria (ARB) and genes (ARGs), the scenario in India, one of the biggest consumers of antibiotics, remains poorly described. In this study, we used metagenomic sequencing to characterize ARGs and biocide/metal resistance genes (BMRGs) in four wastewater treatment plants (WWTPs) in Jaipur City of India. We observed a significantly lower richness and abundance of ARGs in the influent of a WWTP exclusively receiving hospital wastewater when compared to other three WWTPs involving municipal wastewater treatment. Several tetracycline and macrolide-lincosamide-streptogramin resistance genes were enriched in influents of these three municipal wastewater-related treatment plants, whereas hospital wastewater had a higher abundance of genes conferring resistance to disinfectant-related compounds such as synergize and wex-cide-128, reflecting the patterns of antibiotic/disinfectant use. Of note, in the wastewater system with more chemicals, there was a strong correlation between the numbers of ARGs and BMRGs potentially harbored by common hosts. Our study highlights significant influxes of ARGs from non-hospital sources in Jaipur City, and thus more attention should be paid on the emergence of ARGs in general communities.

## Introduction

Antibiotic resistance is a long-standing and ever worsening issue, posing a serious threat to human health worldwide (Rivera-Tapia, 2003; Ventola, 2015; Ben et al., 2019). In addition, antibiotic resistance causes an immense economic burden across the globe (Wang et al., 2018), with clinicians facing a trade-off between prescribing effective antimicrobial medication in practice and preventing further antibiotic resistance. Antibiotic resistance can be co-selected by the antibacterial biocides and metals when bacteria harbor genes conferring resistance to both types of compounds (Pal et al., 2015). Various compounds, including antimicrobial residues, biocides and heavy metals flow into wastewater, further providing a fertile environment for antibiotic-resistant bacteria (ARB) and antibiotic resistance genes (ARGs; Karkman et al., 2018). Therefore, wastewater treatment plants (WWTPs) play a pivotal role in ARGs conservation and dissemination (Barancheshme and Munir, 2018; Wang et al., 2020).

Although WWTPs are originally designed to purify wastewater, they also play a part in removing ARB and ARGs. The ARB/ARGs removal efficiency differs among different WWTPs and is affected by various factors. These include wastewater treatment processes (Kucukunsal and Icgen, 2020; Qin et al., 2020), disinfection methods (Barancheshme and Munir, 2018), the types of ARGs present (Li et al., 2015), and WWTP size (Harnisz et al., 2020). Additionally, a trans-Europe surveillance on antibiotic resistance in WWTPs revealed a correlation between resistance persistence vs. antibiotic use and environmental temperature (Pärnänen et al., 2019). Despite this, the remaining ARGs in WWTP discharges are still concerns for the receiving environments. For example, there is a consensus of findings that the WWTP discharges would increase the ARGs abundance in receiving river (Berglund et al., 2015; Brown et al., 2020).

Typically, the WWTPs collect wastewater from a wide variety of sources, such as residential areas, industry and hospitals. In these sorts of wastewaters, the prevalence of ARGs is expected distinct. While hospital wastewater is considered as an unintentional hotspot of resistome owing to more likely containing antibiotic compounds and patient’s excrement (Rozman et al., 2020), municipal wastewater is also a notable reservoir for ARGs. Limited studies have made an attempt to depict municipal wastewater and hospital wastewater on resistome and/or microbiota. In Kumar’s study, higher prevalence of ARB and ARG were found in hospital WWTP (Kumar et al., 2020). In terms of microbial composition, Ng et al. noticed that hospital wastewater and municipal wastewater influent bore a high similarity (Ng et al., 2017).

A cross-national study in Europe showed higher rates of antibiotic resistance in countries with higher outpatient antibiotic use (Goossens et al., 2005). Since India is one of the largest human antibiotic-consuming countries (Klein et al., 2018), it may have a potentially severe situation in antibiotic resistance. Furthermore, although hospital wastewater reportedly harbors high levels of clinically relevant ARGs (Fouz et al., 2020), the situation might be worse in Indian communities because of a higher prevalence of self-medication that tends to use antibiotics inappropriately (Marathe et al., 2020). Thus far, a few studies using PCR techniques for ARG quantification reported higher load or prevalence of ARGs in hospital wastewater than in municipal wastewater in India (Lamba et al., 2017; Kumar et al., 2020). However, the diversity and abundance of ARGs was likely underestimated due to the limited set of genes targeted, and thus the picture was probably incomplete. In this context, we conducted the first metagenomic survey to characterize and compare the compositions of ARGs and biocide/metal resistance genes (BMRGs) in four functioning WWTPs receiving wastewater from various sources in Jaipur City of India. By characterizing the ARGs and BMRGs in four WWTPs, we attempted to elucidate (1) their profiles in different sites of WWTPs, (2) their difference between wastewater from different sources, and (3) associations between ARGs and BMRGs.

## Materials and Methods

### Study Sites and Sample Collection

Four WWTPs were selected in Jaipur City of India: Malaviya National Institute of Technology Jaipur (MNIT), Jawahar Circle (JC), Jaipur National University of Medical Sciences and Research Centre (JNU), and Eternal Heart Critical Care Hospital (EHCC). EHCC only receives hospital wastewater, whereas the other three WWTPs exclusively or partially treat municipal wastewater. More specifically, MNIT WWTP only treats sewage within the university; JC WWTP primarily receives municipal sewage, but it also receives the discharges from many major hospitals of the city, of which some of them have onsite wastewater treatment facilities such as EHCC and JNU; JNU WWTP is receiving wastewater from the hospital as well as residential colony within the university campus; EHCC WWTP only treats hospital wastewater. A total of 34 samples were collected in the first 2weeks of July 2019 from three different sites (influent, I; sludge, S; and effluent, E) of the four WWTPs. Three biological replicates were performed for each sampling site except for influents of MNIT and EHCC that only had two biological replicates. Detailed characteristics including treatment capacity, types of wastewater treatment process, source of collected wastewater, and disinfection methods are presented in Table 1.

**Table 1:** Characteristics of the four WWTPs.

	MNIT	JC	EHCC	JNU
Treatment capacity	0.5 MLD	1 MLD	0.1 MLD	0.7 MLD
Wastewater treatment process	MBBR	MBBR	MBBR	SBR
Source of collected wastewater	Wastewater from a university	Municipal wastewater (major) Untreated and treated hospital wastewater including effluents from EHCC and JNU (minor)	Hospital wastewater	Hospital wastewater Domestic wastewater within university
Disinfection method	Chlorination	UV irradiation	Chlorination	Chlorination

MLD, millions of liters per day; MBBR, moving bed biofilm reactor; and SBR, sequencing batch reactors.

The samples were collected in 2-liter (I and E) or 1-liter (S) sterile plastic containers. These samples were transported to the laboratory at Birla Institute of Scientific Research (BISR) within 1h after collection and were stored at 4°C until the samples were further processed. A subset of all the samples was prepared by mixing the sample with absolute ethanol (1:1) to inhibit bacterial growth and stored at 4°C.

### DNA Extraction and Quantification

Initially, wastewater samples were filtered through Whatman filter paper (Grade 1, 11μm) to remove any soil traces and precipitates. For influent and effluent samples, genomic DNA was extracted from the filter membrane after filtering approximately 1,000ml of wastewater sample through sterile nylon membranes (0.45μm pore size; 47mm diameter, Pall Life Sciences, United States). Sludge samples (100ml) were centrifuged at 10,000rpm for 30min to separate supernatant and solid residue. Similar to influent and effluent samples, the supernatant fraction was used for membrane filtration. DNA was then extracted from the filter membrane together with ~100mg solid residue of sludge. The DNA from filter membrane for influent and effluent samples or from the pooling of filter membrane and solid residue for sludge sample was extracted using the Fast DNA Spin Kit for Soil (MP Biomedicals, France) per the manufacturer’s instructions. The DNA concentration was quantified using a NanoDropND-1000 spectrophotometer (Thermo Scientific, United States). DNA quality was assessed by agarose gel electrophoresis. DNA samples were stored at −80°C until further analyses.

### Physico-Chemical Analysis of Wastewater

The pH and temperature were monitored using a pH/Temperature tester (pHep, Hanna). Chemical oxygen demand (COD) was measured by the close reflux colorimetric method (APHA et al., 2012) using the HACH-DRB200 digital reactor block. Biochemical oxygen demand (BOD) was estimated by the respirometric method using the HACH BOD Trak system. Total suspended solids (TSS) were measured by the standard method described in APHA (APHA et al., 2012).

### Metagenomic Sequencing

A total of 34 DNA samples including three replicas for each sample (only two replicas for influent samples of EHCC and MNIT) were sent to the Beijing Genomics Institute (BGI) for shotgun metagenomic sequencing with a 100bp paired-end protocol as previously described (Fang et al., 2018). In brief, 1μg of genomic DNA was randomly fragmented by Covaris. The fragmented genomic DNA was then selected by Agencourt AMPure XP-Medium kit and those fragmented genomic DNA with an average size of 200–400bp were used to construct the library. Subsequently, the qualified sequencing library was subjected to 100bp paired-end sequencing using the BGISEQ-500 platform and a dataset with roughly 207Gb of data was generated. The data for this study has been deposited in the European Nucleotide Archive (ENA) at EMBL-EBI under accession number PRJEB38014.^1^

### Identification of ARGs, BMRGs, and Bacterial Community

Raw reads from metagenomic sequencing were processed with Fastp (Chen et al., 2018) for quality control, yielding a dataset with an average of 6.1Gb bases. The cleaned reads were then subjected to ARGs-OAP v2.0 (Yin et al., 2018) for quantifying ARGs and BMRGs. The ARGs reference sequence was swapped for BMRGs sequence obtained from BacMet database (Pal et al., 2014) when quantifying BMRGs. This tool can automatically normalize the abundance of ARGs/BMRGs to the ARGs/BMRGs reference length and the numbers of prokaryote cell. Using ARGs-OAP v2.0, the abundance of ARGs and BMRGs was calculated and expressed as the number of copies of ARGs/BMRGs per cell. Taxonomic classification was conducted by Kraken2 (Wood et al., 2019), in combination with Bracken (Lu et al., 2017) for accurate abundance estimation. When annotating the ARGs/BMRGs, we tagged two attributes (subtype and type) to each ARG/BMRG. The subtype is the gene itself, and the type is the drug (for ARGs) or compound (for BMRGs) to which the gene confers resistance. Only ARGs/BMRGs subtype or species present in ≥2 replicates were retained for downstream analysis.

### Diversity Estimation, Ordination Analysis, and Statistical Analysis

The alpha diversity (Chao1 richness and Shannon diversity) and beta diversity (Bray-Curtis distance) of ARGs and BMRGs were calculated with R package vegan (Oksanen et al., 2020). Principal coordinate analysis (PCoA) of ARG and BMRG beta diversity was done in R. Spearman’s correlation coefficients between bacterial taxa and ARGs/BMRGs were computed with WGCNA (Langfelder and Horvath, 2008), and *p* values were adjusted with the “FDR” method (Benjamini and Hochberg, 1995). A species was regarded as the host of an ARG/BMRG when the Spearman’s correlation coefficient between them >0.6 and adjusted *p* value<0.05. The Welch’s *t*-test was adopted to determine significantly different ARGs and BMRGs between different groups.

## Results

### Physico-Chemical Parameters of Influents and Effluents

An essential role of WWTPs is to remove contaminants from wastewater, in order to minimize the pollution of water courses. Physico-chemical parameters conducted for water quality assessment showed that the influents of three WWTPs with municipal wastewater involvement (MNIT, JC and JNU) had a significantly higher BOD, COD, and TSS (Welch’s *t*-test, *p*<0.05) than the influent of EHCC that exclusively receives hospital wastewater, but this trend was not found in their effluents (Supplementary Table S1).

### Overall Profiles of ARGs and BMRGs

**Figure 1:**
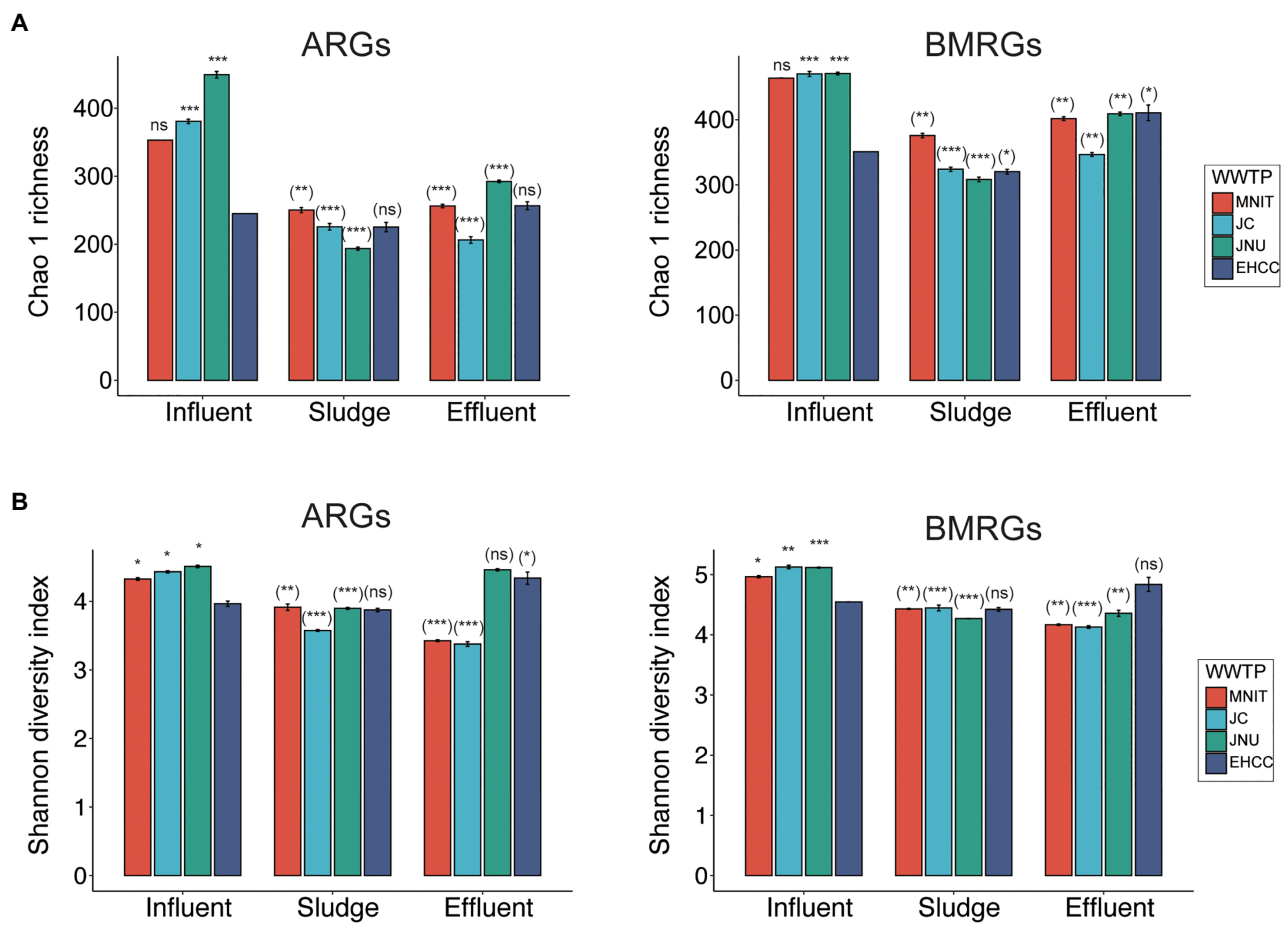
Alpha diversity of ARGs and BMRGs. Bar diagrams show the chao1 richness **(A)** and Shannon diversity **(B)** of ARGs and BMRGs based on ARGs/BMRGs subtypes in influents, sludges, and effluents of four WWTPs. Data are mean±standard deviation. The Chao1 richness of ARGs/BMRGs for influents of MNIT or EHCC were identical in two replicas, therefore the standard deviations for them were zero. Significances between the influents of MNIT/JC/JNU vs. influent of EHCC (outside brackets) and between influent and sludge/effluent for each WWTP (inside brackets) were indicated by using Welch’s *t*-test. ^*^p<0.05; ^**^p<0.01; ^***^p<0.001; ns, not significant.

**Figure 2:**
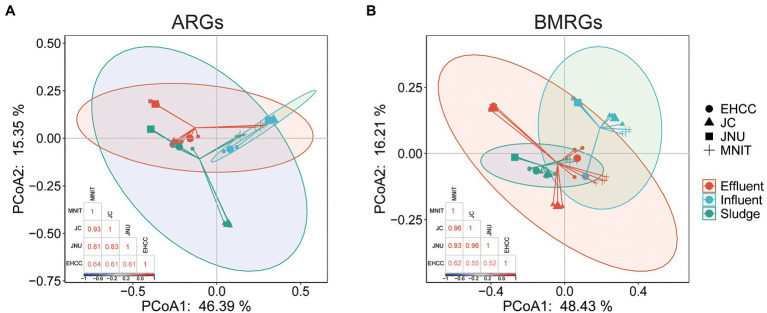
Principal coordinate analysis (PCoA) of ARGs **(A)** and BMRGs **(B)** and heatmap of Spearman correlation among influents of four WWTPs. Both analyses were based on ARGs/BMRGs subtypes.

**Figure 3:**
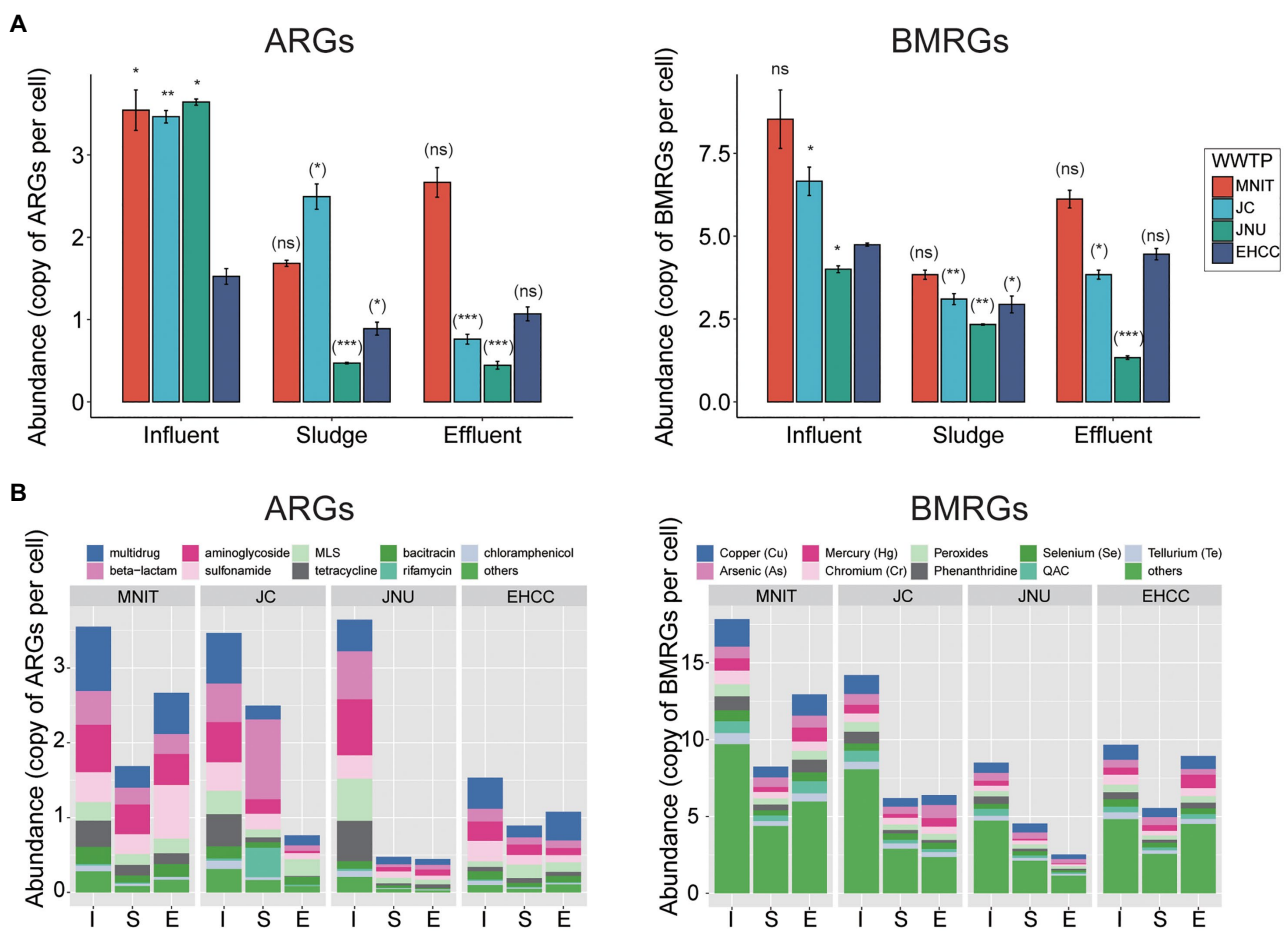
The absolute abundance of ARGs and BMRGs based on subtypes **(A)** or types **(B)**. Data in **(A)** are mean±standard deviation. Significances between the influents of MNIT/JC/JNU vs. influent of EHCC (outside brackets) and between influent and sludge/effluent for each WWTP (inside brackets) were indicated by using Welch’s *t*-test. ^*^p<0.05; ^**^p<0.01; ^***^p<0.001; ns, not significant. The mean abundance was presented for each sampling sites in **(B)**. Top nine prevalent types were showed, whereas the rest types were clustered into others. I, Influent; S, Sludge; E, Effluent; MLS, macrolide-lincosamide-streptogramin; and QACs, quaternary ammonium compounds.

Likewise, the richness (Chao1) and biodiversity (Shannon) of both ARGs and BMRGs were significantly higher in the influents of these three WWTPs than the influent of EHCC (Welch’s *t*-test, *p*<0.05; Figure 1). After treatment, as compared to the respective influents, the richness of ARGs and BMRGs significantly decreased in sludges and effluents of MNIT, JC, and JNU, but not EHCC (Figure 1A). Interestingly, ARGs and BMRGs in the influents of MNIT, JC, and JNU were substantially similar (Spearman’s *rho*>0.8, *p*<0.05) despite these treatment plants receive wastewater from various sources, whereas they became more diverse in effluents (Figure 2; Supplementary Figure S1). Moreover, the influents of MNIT, JC, and JNU had a significantly higher abundance of ARGs than influent of EHCC (average 3.55, 3.47, 3.64 vs. 1.53, *p*<0.05). As for BMRGs, the abundance in influents of MNIT and JC was higher than JNU or EHCC (average 8.53, 6.66 vs. 4.0, 4.74; Figure 3A). The wastewater treatment in JC and JNU significantly reduced the abundance of ARGs and BMRGs from influent to effluent. At the type level, genes conferring resistance to multidrug (ARGs, ranging 0.06–0.98 copy/cell) and to copper (BMRGs, ranging 0.30–1.99 copies/cell) were the most abundant (Figure 3B).

### Discriminative ARGs and BMRGs Among Influents

**Figure 4:**
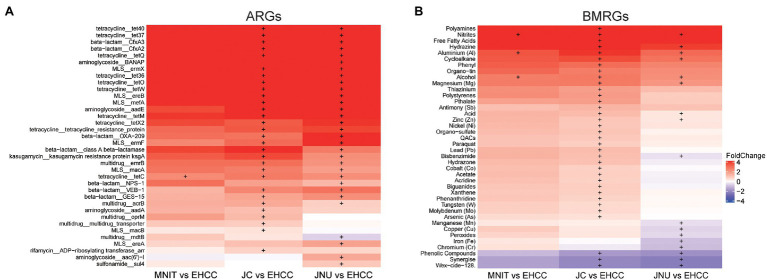
Differentially abundant ARGs **(A)** and BMRGs **(B)** among influents. Heat map illustrates the fold change of ARGs/BMRGs abundance between influents of MNIT/JC/JNU vs. influent of EHCC. Positive fold change means higher abundance of ARGs/BMRGs in influents of MNIT/JC/JNU, otherwise ARGs/BMRGs enrich in influent of EHCC. The significance computed by Welch’s *t*-test was shown with plus sign. Only the ARGs subtypes or BMRGs types with average abundance>0.01 copy per cell, and with significantly differential abundance in no less than one comparison was shown. MLS, macrolide-lincosamide-streptogramin; QACs, quaternary ammonium compounds.

Since influents include wastewaters resulting from various human activities, we then compared the abundance of individual ARGs and BMRGs among them. Several ARGs related to tetracycline, beta−lactam, and macrolide-lincosamide-streptogramin (MLS) were found enriched in MNIT, JC, and JNU compared to EHCC (Figure 4A). In addition, these three WTTPs also had a significantly higher abundance of BMRGs conferring resistance to various compounds such as aluminum and alcohol. Conversely, the abundance of BMRGs related to phenolic compounds, synergize compound, and wex−cide−128 compound were higher in EHCC (Figure 4B).

### Common Host of ARGs and BMRGs

**Figure 5:**
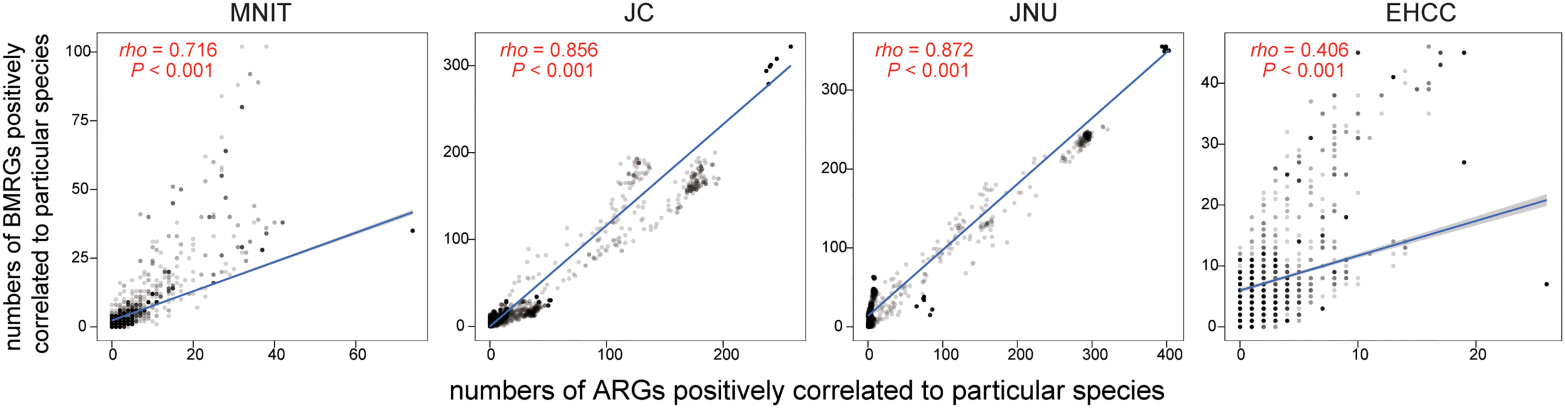
The correlation between the number of ARGs and the number of BMRGs, in which ARGs and BMRGs are positively correlated to one species with Spearman’s correlation coefficient>0.6. Each dot corresponds to one bacterial species that is regarded as the host of ARGs and BMRGs. Spearman’s correlation coefficient between them and *p* value are shown in red.

Finally, we explored the bacterial community and their relationship to ARGs/BMRGs. The abundance for the top four phyla are as follows: Proteobacteria (ranging from 50.75 to 89.79%), Bacteroidetes (ranging from 0.57 to 36.97%), Actinobacteria (ranging from 1.13 to 13.49%), and Firmicutes (ranging from 0.52 to 19.68%; Supplementary Figure S2). We found intestinal bacteria presenting more abundantly in the influents of MNIT, JC, and JNU than the influent of EHCC (Supplementary Table S2). By estimating the numbers of ARGs and BMRGs potentially carried by common hosts (4,995 species in total), we observed stronger correlations between these numbers (*rho*=0.72, 0.86, 0.87) in MNIT, JC, and JNU compared to that of EHCC (*rho*=0.41; Figure 5).

## Discussion

Hospital wastewater is believed to contain more pharmaceutically active compounds than municipal wastewater, as well as the excrement of hospitalized patients who are more likely to harbor bacteria rich in ARGs. Indeed, a previous study showed hospital wastewater presenting more types and higher abundance of ARGs than those in most wastewater systems (Zhang et al., 2020). However, using metagenomics which enables more comprehensive profiling of the resistomes, we found opposite trends in this study (Figures 1, 3). Lower abundance of gut bacteria indicates that the influent of EHCC has fewer fecal content, further minimizing the risk of ARGs enrichment. This can be corroborated by a previous study where fecal pollution can largely explain the presence of ARGs in the environment (Karkman et al., 2019). Furthermore, excessive over-the-counter antibiotic use in community pharmacies, likely leading to inappropriate use of antibiotics, is another concern in India (Gong et al., 2020). Both phenomena underline municipal wastewater as being a vital source for ARGs.

As compared to hospital wastewater, the influents of other WWTPs have an elevated abundance of ARGs related to tetracycline and MLS (Figure 4). Tetracycline use is limited in human medicine nowadays, but it is widely used in the treatment of livestock and aquaculture (Pazda et al., 2019). Previous studies also reported lower consumption of macrolides in the hospital sector than communities (Ecdc, 2018; Pazda et al., 2019). These practices, to some extent, explain the enriched tetracycline and MLS resistant genes found in municipal wastewater. In BMRGs terms, genes resistant to various compounds were enriched in influents of three WWTPs receiving municipal wastewater, especially in JC. Physico-chemical parameters reflect more chemicals in these WWTPs (Supplementary Table S1). Most chemicals such as metals in wastewater are not necessary to bacterial growth; on the contrast, they pose a selective pressure on bacterial survival. Bacteria tend to evolve and become resistant through various mechanisms to survive these chemicals. Therefore, more chemicals present in these three WWTPs might pose a more intensive pressure to bacteria, and further contribute to the enrichment of BMRGs. On the other hand, phenolic compounds, synergize, and wex-cide-128 resistance genes were more abundant in hospital wastewater. Synergize (mainly containing quaternary ammonium chloride and glutaraldehyde) and wex-cide-128 (mainly including 2-phenylphenol) are commonly used disinfectants nowadays. Perhaps the intensive use of these compounds in hospitals results in the high abundance of genes conferring resistance to these compounds, which attributed to those (gene *adeA*, *adeB*, *adeC*, *adeI*, *adeT1*, and *adeT2*) coding for efflux pump.

From an evolutionary perspective, co-selection with BMRGs is a driving force of ARG spread that deserves notice in the post-antibiotic era. Pal et al. (2015) provided the evidence that bacteria harboring BMRGs more frequently carried ARGs than those without, which was in part consistent with our findings. We observed the number of ARGs was strongly correlated to the number of BMRGs harbored by the potentially same host in three WWTPs but not hospital WWTP EHCC (Figure 5). A large number of matters in three WWTPs with municipal wastewater collection might promote the co-occurrence of ARGs and BMRGs. Meanwhile, horizontal gene transfer can be accelerated under the stress provoked by pollutant compounds in wastewater (Aminov, 2011), which increases the exchange of ARGs and BMRGs among the bacterial community. From the standpoint of waste disposal, municipal wastewater might contain more diverse wastes resulting from various human activities when compared to hospital wastewater. Consequently, the ARGs have less associations with BMRGs in hospital WWTP.

This study is limited by the lack of information on loads of influx from different sources for each WWTP, composition of chemical compounds including antibiotic residues in influents, population number the four WWTPs served, and antibiotic consumption in communities and hospitals generating the wastewater surveyed. Despite these limitations, our metagenomic survey shows for the first time, that municipal wastewater has more diverse and abundant ARGs and BMRGs than hospital wastewater. These findings emphasize that municipal wastewater may play a more important role in ARG/ARB transmission than previously perceived, and thus, deserves careful surveillance in Indian. Future studies are warranted to confirm the driving force of these differences.

## Conclusion

This study showed that the selected representatives of gut bacteria enriched in influents of three WWTPs with municipal wastewater collection when compared to the influent of hospital WWTP. This, together with prevailing over-the-counter antibiotic dispensing in communities, might contribute to the higher types and abundance of ARGs observed in municipal wastewater, suggesting its significant role in monitoring antibiotic resistance in India. The close association between ARGs and BMRGs in wastewater containing more pollutant compounds underscores the potential risk of ARGs dissemination and survival of antibiotic-resistant pathogens.

## Data Availability Statement

The datasets presented in this study can be found in online repositories. The names of the repository/repositories and accession number(s) can be found at: https://www.ebi.ac.uk/ena, PRJEB38014.

## Author Contributions

HT: conceptualization, supervision, project administration, and funding acquisition. C-LC and HO: methodology. DZ: software, data curation, and writing – original draft preparation. DZ, YP, SL, and HT: formal analysis. SS, AG, AV, and RC: investigation and resources. DZ, YP, HK-FW, XZ, YX, KM, KF, and HT: writing – review and editing. All authors contributed to the article and approved the submitted version.

## Conflict of Interest

The authors declare that the research was conducted in the absence of any commercial or financial relationships that could be construed as a potential conflict of interest.

## Publisher’s Note

All claims expressed in this article are solely those of the authors and do not necessarily represent those of their affiliated organizations, or those of the publisher, the editors and the reviewers. Any product that may be evaluated in this article, or claim that may be made by its manufacturer, is not guaranteed or endorsed by the publisher.

## Footnotes

^1^ https://www.ebi.ac.uk/ena/data/view/PRJEB38014
