# Supplementary material for: Metagenomic Survey Reveals More Diverse and Abundant Antibiotic Resistance Genes in Municipal Wastewater Than Hospital Wastewater
Source: Front Microbiol. 2021 Aug 30;12:712843. doi: 10.3389/fmicb.2021.712843 (PMC8435860; doi:10.3389/fmicb.2021.712843)
Supplement: Supplementary file 1 [file Data_Sheet_1.DOCX]

Supplementary Material

# Supplementary Figures and Tables

## Supplementary Figures

**
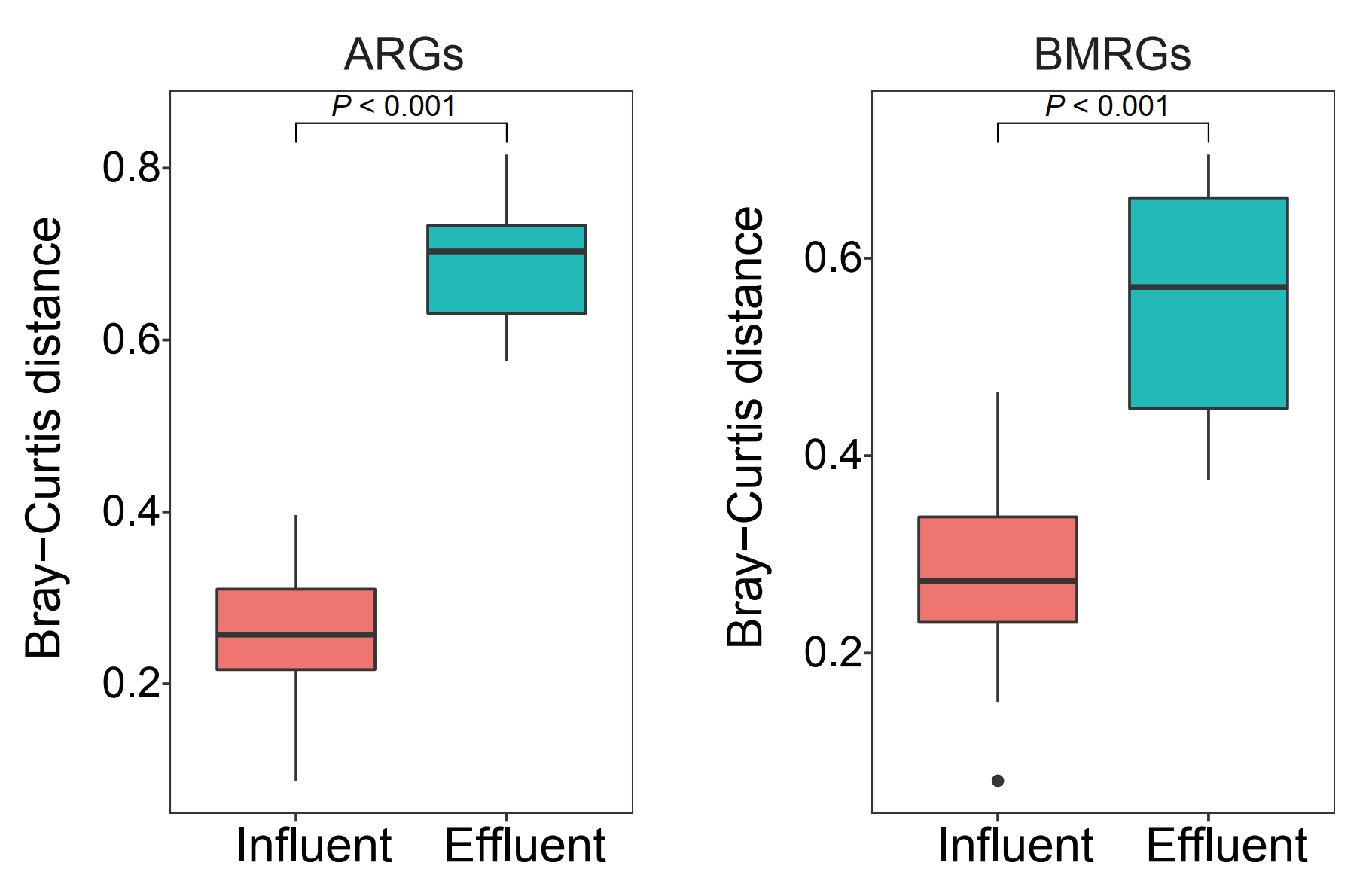
**

**Supplementary Figure S1.** Bray-Curtis dissimilarity between the influents and effluents of three WWTPs (MNIT, JC and JNU) in ARGs/BMRGs. The distance between the replicates for each sample was excluded. Mann–Whitney U test was adopted to compute the significance.

**
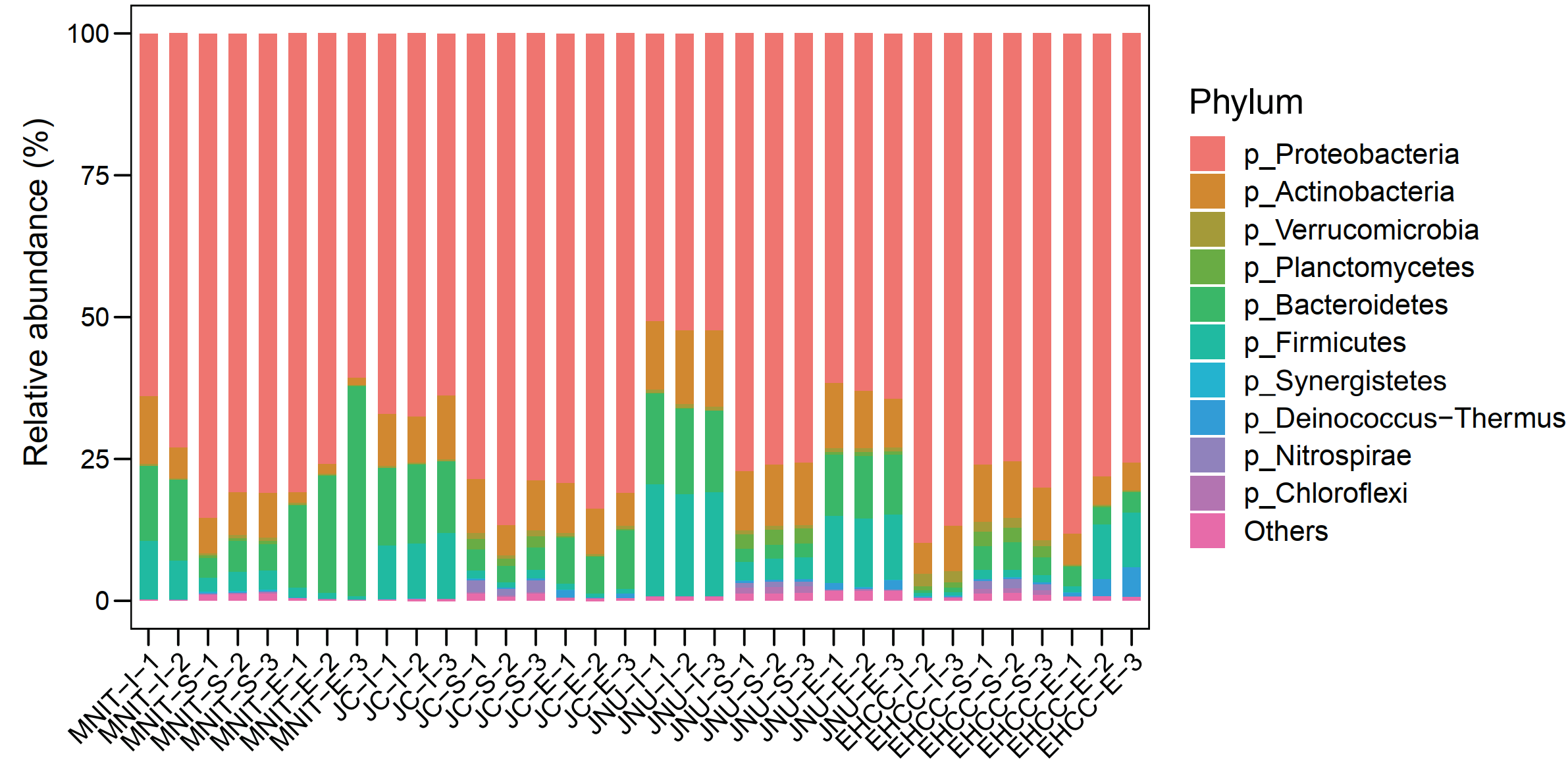
**

**Supplementary Figure S2.** The profile of microbial composition. Bar plot shows the relative abundance of bacterial taxa at phylum level. The phyla with abundance < 1% in all samples were included into others.

## Supplementary Tables

**Supplementary Table S1. Physico-chemical parameters of the raw and treated wastewater.**

| **WWTP** | **Site** | **pH** | **Temp (°C)** | **BOD (mg/L)** | **COD (mg/L)** | **TSS (mg/L)** |
| --- | --- | --- | --- | --- | --- | --- |
| MNIT | Influent | 6.94 ± 0.144 | 27.83 ± 0.57 | 167.33 ± 4.04* | 495.00 ± 13.22* | 187.00 ± 6.00* |
|  | Effluent | 7.56 ± 0.05 | 26.63 ± 0.23 | 28.33 ± 1.25 | 64.36 ± 1.97 | 29.6 ± 1.68 |
| JC | Influent | 6.72 ± 0.06 | 26.9 ± 0.36 | 366.00 ± 12.00*** | 1092.66 ± 8.62*** | 306.00 ± 5.00*** |
|  | Effluent | 7.60 ± 0.10* | 27.60 ± 0.55 | 11.03 ± 0.45 | 35.03 ± 1.30* | 29.50 ± 1.32 |
| JNU | Influent | 7.80 ± 0.04 | 28.00 ± 0.47 | 189.00 ± 6.01*** | 646.67 ± 6.40* | 186.00 ± 6.06** |
|  | Effluent | 7.39 ± 0.03 | 27.50 ± 0.56 | 18.18 ± 1.55 | 60.80 ± 0.26* | 61.10 ± 1.30* |
| EHCC | Influent | 7.65 ± 0.12 | 27.50 ± 0.39 | 44.00 ± 1.55 | 258.00 ± 7.50 | 69.00 ± 5.00 |
|  | Effluent | 7.24 ± 0.07 | 26.70 ± 0.42 | 21.60 ± 1.12 | 90.30 ± 2.30 | 10.00 ± 2.30 |

Data are mean ± standard deviation. *P* values were given by Welch's *t*-test. “*” showed the significance between the influent of MNIT/JC/JNU and influent of EHCC. “*” showed the significance between the effluent of MNIT/JC/JNU and effluent of EHCC. *: *P* < 0.05; **: *P* < 0.01; ***: *P* < 0.001. Temp: Temperature; BOD: Biochemical Oxygen Demand; COD: Chemical Oxygen Demand; TSS: Total suspended solids.

| **Phylum** | **Genus^1^** | **Abundance (%)** | | | |
| --- | --- | --- | --- | --- | --- |
|  |  | **MNIT** | **JC** | **JNU** | **EHCC** |
| Bacteroidetes | *Bacteroides* | 5.38 ± 0.52 | 6.36 ± 0.54** | 8.79 ± 0.56** | 0.06 ± 0.01 |
|  | *Prevotella* | 0.16 ± 0.01* | 0.54 ± 0.09* | 1.14 ± 0.07** | 0.01 ± 0.01 |
|  | *Xylanibacter* | ND | ND | ND | ND |
| Firmicutes | *Ruminococcus* | 0.07 ± 0.01 | 0.07 ± 0.01* | 0.22 ± 0.03** | 0 ± 0 |
|  | *Clostridium* | 0.03 ± 0 | 0.07 ± 0.01* | 0.13 ± 0.01*** | 0.03 ± 0 |
|  | *Lactobacillus* | 0.78 ± 0.25 | 0.97 ± 0.12** | 1.67 ± 0.15** | 0.02 ± 0 |
|  | *Roseburia* | 0.29 ± 0.04 | 0.26 ± 0.02** | 0.77 ± 0.02*** | 0.01 ± 0 |
|  | *Eubacterium* | 0.01 ± 0 | 0.02 ± 0 | 0.02 ± 0 | ND |
|  | *Faecalibacterium* | 1.63 ± 0.16 | 1.07 ± 0.19* | 4.36 ± 0.21** | 0.02 ± 0 |
| Actinobacteria | *Collinsella* | 1.09 ± 0.39 | 1.03 ± 0.12** | 1.25 ± 0.08** | 0.03 ± 0 |
|  | *Bifidobacterium* | 7.01 ± 2.76 | 7.75 ± 1.13* | 11.18 ± 0.51** | 0.16 ± 0.01 |
| Proteobacteria | *Desulfovibrio* | 0.16 ± 0.03 | 0.52 ± 0.11* | 0.41 ± 0.02** | 0.17 ± 0 |
| Verrucomicrobia | *Akkermansia* | 0.14 ± 0.03 | 0.23 ± 0.03** | 0.49 ± 0.04** | 0.01 ± 0 |

**Supplementary Table S2. The abundance of representatives of intestinal bacteria in the influents of four WWTPs.**

^1^A list reported in [1].

Data are mean ± standard error. ND: not detected. Significance between either of MNIT, JC, and JNU versus EHCC was given by Welch's *t*-test. *: *P* < 0.05; **: *P* < 0.01; ***: *P* < 0.001.

**Reference:**

1. Kushkevych I, Martínková K, Vítězová M, Rittmann SK-MR. Intestinal Microbiota and Perspectives of the Use of Meta-Analysis for Comparison of Ulcerative Colitis Studies. J Clin Med. 2021.
